# Fecal Lipocalin 2, a Sensitive and Broadly Dynamic Non-Invasive Biomarker for Intestinal Inflammation

**DOI:** 10.1371/journal.pone.0044328

**Published:** 2012-09-05

**Authors:** Benoit Chassaing, Gayathri Srinivasan, Maria A. Delgado, Andrew N. Young, Andrew T. Gewirtz, Matam Vijay-Kumar

**Affiliations:** 1 Georgia State University, Department of Biology, Center for Inflammation, Immunity and Infection, Atlanta, Georgia, United States of America; 2 Emory University School of Medicine, Department of Pathology, Atlanta, Georgia, United States of America; 3 Grady Medical Center, Atlanta, Georgia, United States of America; Charité-University Medicine Berlin, Germany

## Abstract

Inflammation has classically been defined histopathologically, especially by the presence of immune cell infiltrates. However, more recent studies suggest a role for "low-grade" inflammation in a variety of disorders ranging from metabolic syndrome to cancer, which is defined by modest elevations in pro-inflammatory gene expression. Consequently, there is a need for cost-effective, non-invasive biomarkers that, ideally, would have the sensitivity to detect low-grade inflammation and have a dynamic range broad enough to reflect classic robust intestinal inflammation. Herein, we report that, for assessment of intestinal inflammation, fecal lipocalin 2 (Lcn-2), measured by ELISA, serves this purpose. Specifically, using a well-characterized mouse model of DSS colitis, we observed that fecal Lcn-2 and intestinal expression of pro-inflammatory cytokines (IL-1β, CXCL1, TNFα) are modestly but significantly induced by very low concentrations of DSS (0.25 and 0.5%), and become markedly elevated at higher concentrations of DSS (1.0 and 4.0%). As expected, careful histopathologic analysis noted only modest immune infiltrates at low DSS concentration and robust colitis at higher DSS concentrations. In accordance, increased levels of the neutrophil product myeloperoxidase (MPO) was only detected in mice given 1.0 and 4.0% DSS. In addition, fecal Lcn-2 marks the severity of spontaneous colitis development in IL-10 deficient mice. Unlike histopathology, MPO, and q-RT-PCR, the assay of fecal Lcn-2 requires only a stool sample, permits measurement over time, and can detect inflammation as early as 1 day following DSS administration. Thus, assay of fecal Lcn-2 by ELISA can function as a non-invasive, sensitive, dynamic, stable and cost-effective means to monitor intestinal inflammation in mice.

## Introduction

The two major chronic inflammatory bowel diseases (IBD), Crohn’s Disease (CD) and Ulcerative Colitis (UC), are characterized by acute to chronic inflammation of the gastrointestinal tract affecting over a million individuals in the United States [1]. Although IBD etiology is obscure, significant evidence suggest that inflammation results from aberrant immune responses to gut microbiota in genetically susceptible host, which is also influenced by other environmental triggers [2], [3]. IBD patients are also at the potential risk of developing intestinal neoplasias [4]. Currently, clinical diagnosis of IBD is achieved through endoscopic appearance and basic laboratory markers although cost, invasiveness, and lack of specificity are acknowledged limitations [5]. Therefore there is a need for identification of potential sensitive and non-invasive markers that can facilitate diagnosis before the onset of clinical symptoms. In addition, it is increasingly appreciated that low-grade chronic inflammation in the gut can also promote metabolic disorders such as type II diabetes, atherosclerosis and metabolic syndrome [6], [7]. Thus, improved identification of low-grade gut inflammation might be potentially useful in researching, diagnosing, and/or treating IBD and metabolic diseases.

Lipocalin 2 (Lcn-2) and its human counter part neutrophil gelatinase-associated lipocalin (NGAL) belong to a family of small, secreted proteins expressed by a variety of cells, the richest source being neutrophils [8]. Results from our laboratory and others have shown systemic upregulation of Lcn-2 (also known as siderocalin, uterocalin and 24p3) in various murine models of colitis [9]–[11], including human IBD [12]. In addition, human NGAL has been reported to be increased in patients with ulcerative colitis [13]. Recently, it has been shown that Lcn-2 is real-time marker for renal diseases and meets all the criteria required for a biomarker [14], [15]. The pro-inflammatory transcription factor NF-κB transactivates Lcn-2 expression by binding to the consensus motif within its promoter [16]–[18]. Expression of Lcn-2 in adipose tissue is elevated in various experimental models of obesity and in obese humans, suggesting that Lcn-2 may participate in inflammation-related disorders [19]–[23].

In this study we investigated the extent to which fecal Lcn-2 can serve as a sensitive and non-invasive biomarker of intestinal inflammation using a well-studied murine models of dextran sulfate sodium (DSS) induced colitis and spontaneous colitis in IL-10 deficient mice. Our results demonstrate that fecal Lcn-2 levels provide a stable, rapid, sensitive and broadly-dynamic means to non-invasively detect both low-grade inflammation and robust (*i.e.* classic) colitis.

## Methods

### Mice

Eight weeks old male C57BL/6 and female IL-10 KO mice were purchased from Jackson Laboratories. Mice were housed in specific pathogen free conditions and fed *ad libitum.* Lcn-2KO mice generated by Dr. Shizuo Akira (Japan) were obtained via Dr. Alan Aderem (University of Washington).

### Ethics Statement

Animal protocols were approved by institutional animal care and use committee (Georgia State University Atlanta, GA), permit number A11024.

### DSS Induced Acute-colitis

Mice were administered DSS (MP Biomedicals, Solon, OH) at 0.25, 0.5, 1.0 and 4.0% in drinking water *ad libitum* for 7 days (5 mice per group). Control mice were given water only. During this period, mice were weighed and feces were collected every day and frozen at −20°C. After 7 days, mice were bled *via* retroorbital plexus and hemolysis-free serum was collected by centrifugation using serum separator tubes (BD Biosciences, Franklin Lakes, NJ). Mice were sacrificed by CO_2_ euthanasia. Spleen and colon weights and lengths were measured. A small piece (50 mg) of proximal colon was taken for MPO analysis and RNA extraction, and the rest of the colon fixed in 10% buffered formalin for histological studies. For fecal Lcn-2 expression during mucosa healing, mice were given 1.5% DSS in drinking water for 7 days followed by 29 days of water without DSS prior to analysis of fecal Lcn-2.

### Fecal Lcn-2 in IL-10 KO Spontaneous Colitis

Eight-week-old (n = 20) female IL-10 KO mice and their BL6 WT controls were procured from Jackson Laboratories, housed in GSU’s animal facility, and assayed for fecal Lcn-2 by ELISA after 12 weeks.

### Quantification of Fecal and Serum Lcn-2 by ELISA

Freshly collected or frozen fecal samples were reconstituted in PBS containing 0.1% Tween 20 (100 mg/ml) and vortexed for 20 min to get a homogenous fecal suspension. These samples were then centrifuged for 10 min at 12,000 rpm and 4°C. Clear supernatants were collected and stored at −20°C until analysis. Lcn-2 levels were estimated in the supernatants using Duoset murine Lcn-2 ELISA kit (R&D Systems, Minneapolis, MN). Serum samples were diluted in kit-recommended reagent diluent (1.0% BSA in PBS). Similarly treated Lcn-2KO mouse feces and serum (control and DSS-treated mice) were used as negative controls. For fecal Lcn-2 stability studies, freshly collected feces were either frozen at −20°C or 4°C, stored at room temperature for 24 h, or boiled for 1 h at 99°C before being assayed.

### qRT-PCR

Total RNA was isolated from colonic tissues using TRIzol (Invitrogen, Carlsbad, CA) according to the manufacturer’s instructions. RNA were purified via precipitation with lithium chloride, and quantitative RT-PCR were performed using the Qiagen kit QuantiFast® SYBR® Green RT-PCR in a CFX96 apparatus (Bio-Rad, Hercules, CA) with specific mouse oligonucleotides. The sense and antisense oligonucleotides used were, respectively, the following: 36B4 5′-TCCAGGCTTTGGGCATCA-3′ and 5′-CTTTATTCAGCTGCACATCACTCAGA-3′, TNF-α 5′-ACTCCAGGCGGTGCCTATGT-3′ and 5′-AGTGTGAGGGTCTGGGCCAT-3′, KC 5′-TTGTGCGAAAAGAAGTGCAG-3′ and 5′-TACAAACACAGCCTCCCACA-3′, IL-1β 5′-TTGACGGACCCCAAAAGATG-3′ and 5′-AGAAGGTGCTCATGTCCTCAT-3′and Lcn-2 5′-AAGGCAGCTTTACGATGTACAGC-3′ and 5′-CTTGCACATTGTAGCTGTGTACC-3′. Results were normalized to the housekeeping 36B4 gene.

### Cytokine Analysis

Serum Keratinocyte derived-chemokine (KC) concentration were determined using Duoset cytokine ELISA kits (R&D Systems, Minneapolis, MN) according to manufacturer’s instructions.

### Serum Lcn-2 Immunoblotting

Immunoblotting was performed as described [10]. Briefly, serum was diluted (1∶10) in 2X Laemmli sample buffer (Bio-Rad, Hercules, CA) with β-mercaptoethanol, boiled for 5 min and immediately cooled on ice for 10 min before being subjected to SDS-PAGE on a 4–20% gel (Bio-Rad, Hercules, CA). Proteins were transferred to nitrocellulose, blocked with 5% milk for 1 h, probed with biotinylated anti-mouse Lcn-2 (0.2 ug/ml, R&D Systems, Minneapolis, MN) overnight at 4°C, washed 3x and probed with streptavidin HRP (Invitrogen, Carlsbad, CA) for 1 h at RT. Immunoblots were developed by ECL (GE Healthcare, Buckinghamshire, UK).

### Colonic Myeloperoxidase (MPO) Assay

Neutrophil influx in tissue was analyzed by assaying the enzymatic activity of MPO, a marker for neutrophils. Briefly, tissue (50 mg/mL) was thoroughly washed in PBS and homogenized in 0.5% hexadecyltrimethylammonium bromide (Sigma, St. Louis, MO) in 50 mM PBS, (pH 6.0), freeze-thawed 3 times, sonicated and centrifuged. MPO was assayed in the clear supernatant by adding 1 mg/mL of dianisidine dihydrochloride (Sigma, St. Louis, MO) and 5×10^−4%^ H_2_O_2_ and the change in optical density measured at 450 nm. Human neutrophil MPO (Sigma, St. Louis, MO) was used as standard. One unit of MPO activity was defined as the amount that degraded 1.0 µmol of peroxide/min at 25°C [24].

### H&E Staining of Colonic Tissue and Histopathologic Analysis

Following euthanasia, mouse colons were fixed in 10% buffered formalin for 24 hours at room temperature and then embedded in paraffin. Tissues were sectioned at 5-µm thickness and stained with hematoxylin & eosin (H&E) using standard protocols. H&E stained slides were scored by two pathologists (A.N.Y. and M.A.D.). Each colon was assigned four scores based on the degree of epithelial damage and inflammatory infiltrate in the mucosa, submucosa and muscularis/serosa, as previously described [25]. A slight modification was made to this scoring system; each of the four scores was multiplied by 1 if the change was focal, 2 if it was patchy and 3 if it was diffuse. The 4 individual scores per colon were added, resulting in a total scoring range of 0–36 per mouse. The scores for each of five mice per treatment group (i.e., exposed to 0, 0.25, 0.5, 1.0 and 4.0% DSS) were averaged.

### Statistics

Statistical analysis for significance (p<0.05) was determined using Student’s t-test (GraphPad Prism). Differences were noted as significant * p<0.05.

## Results

### DSS-induced Gross Pathology is dose Dependent

**Figure 1:**
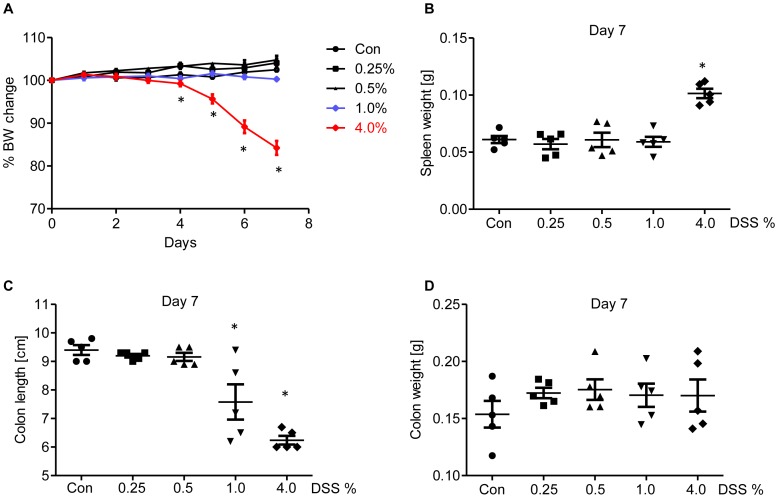
DSS induced gross pathology is dose dependent. Eight week old male BL6 mice were administered DSS (0, 0.25, 0.5, 1.0, and 4.0%) in drinking water for 7 days and monitored every day (5 mice per group). **A**, Loss in body weight; **B**, spleen weight; **C**, colon length and **E**, Colon weight. * p<0.05.

The goal of this study was to investigate the utility of fecal Lcn-2 to serve as a non-invasive biomarker of intestinal inflammation. Intestinal inflammation was induced by giving mice drinking water with 1.0% and 4.0% DSS, which numerous studies have shown induce moderate but easily appreciable and robust colitis, respectively. Moreover, to attempt to induce low-grade inflammation, mice were given doses of 0.25 and 0.5% DSS, both well below those typically used to induce colitis in C57BL/6 mice. As shown in **figure 1A**, after 7 days of DSS administration, the group which received 4.0% DSS exhibited significant loss in body weights, a hallmark feature of this model. Mice which were given 0.5 and 1.0% DSS showed modest decreases in body weight and the group given 0.25% DSS is almost comparable to the control group that received only water. Accordingly, the 4.0% DSS group also exhibited other characteristic symptoms of DSS-induced colitis, such as significant decrease in colon length and modest increase in colon weight as well as splenomegaly (**Figure 1** **B–D**), a feature associated with anemia due to extensive rectal bleeding.

### DSS Dose Dependent Elevation of Colonic and Systemic Pro-inflammatory Gene Expression

**Figure 2:**
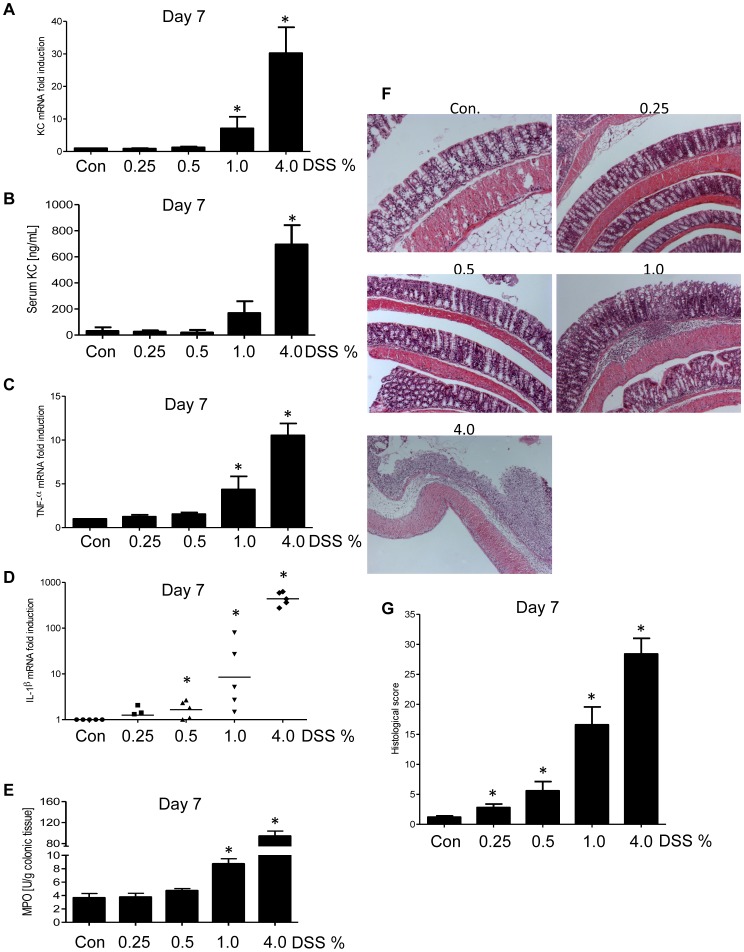
DSS dose dependent increase in pro-inflammatory gene expression and histological damages. Colonic pro-inflammatory cytokine-encoding genes were quantified by qRT-PCR (**A**, KC; **C**, TNF-α; **D**, IL-1β) and KC was quantified in the serum by ELISA (**B**). Colonic MPO were determined (**E**), and H & E stained sections of the colon (**F**) were used for the determination of the histopathological score (**G**). * p<0.05.

Next, we analyzed pro-inflammatory gene expression in the colon by qRT-PCR and serum cytokines by ELISA. In mice given 4.0% DSS, colonic KC levels were significantly upregulated at the mRNA level (30-fold, **Figure 2A**), which was reflected by concomitant elevation in systemic (*i.e.* serum) levels of the neutrophil chemoattractant KC/CXCL1 (**Figure 2B**). KC levels, a key mediator of neutrophil infiltration in the intestinal mucosa, were modestly elevated in mice given 1.0% DSS whereas no change in KC levels was observed either at the mRNA or protein level in mice given lower doses of DSS (**Figure 2** **A–B**). A similar trend was observed for mRNA levels of the potent pro-inflammatory cytokines TNF-α and IL-1β in the colon (**Figure 2** **C–D**). In accordance with colonic and systemic KC levels, MPO levels were significantly upregulated in the 4.0% DSS-treated group, but only modestly elevated in mice given 1.0% and lower doses of DSS (**Figure 2E**). Careful histological scoring of colonic tissue, performed in a blinded fashion by 2 clinical pathologists (A.N.Y. and M.A.D.) detected modest evidence of inflammation in response to 0.25 and 0.5% DSS, whereas mice given 1.0% DSS exhibited clear indications of immune cell infiltration accompanied by epithelial ulceration and sub-mucosal thickening (**Figure 2F–G**). As expected, mice given 4.0% DSS displayed classic robust colitis marked by loss of epithelia and severe immune cell infiltration. (**Figure 2F–G**).

**Figure 3:**
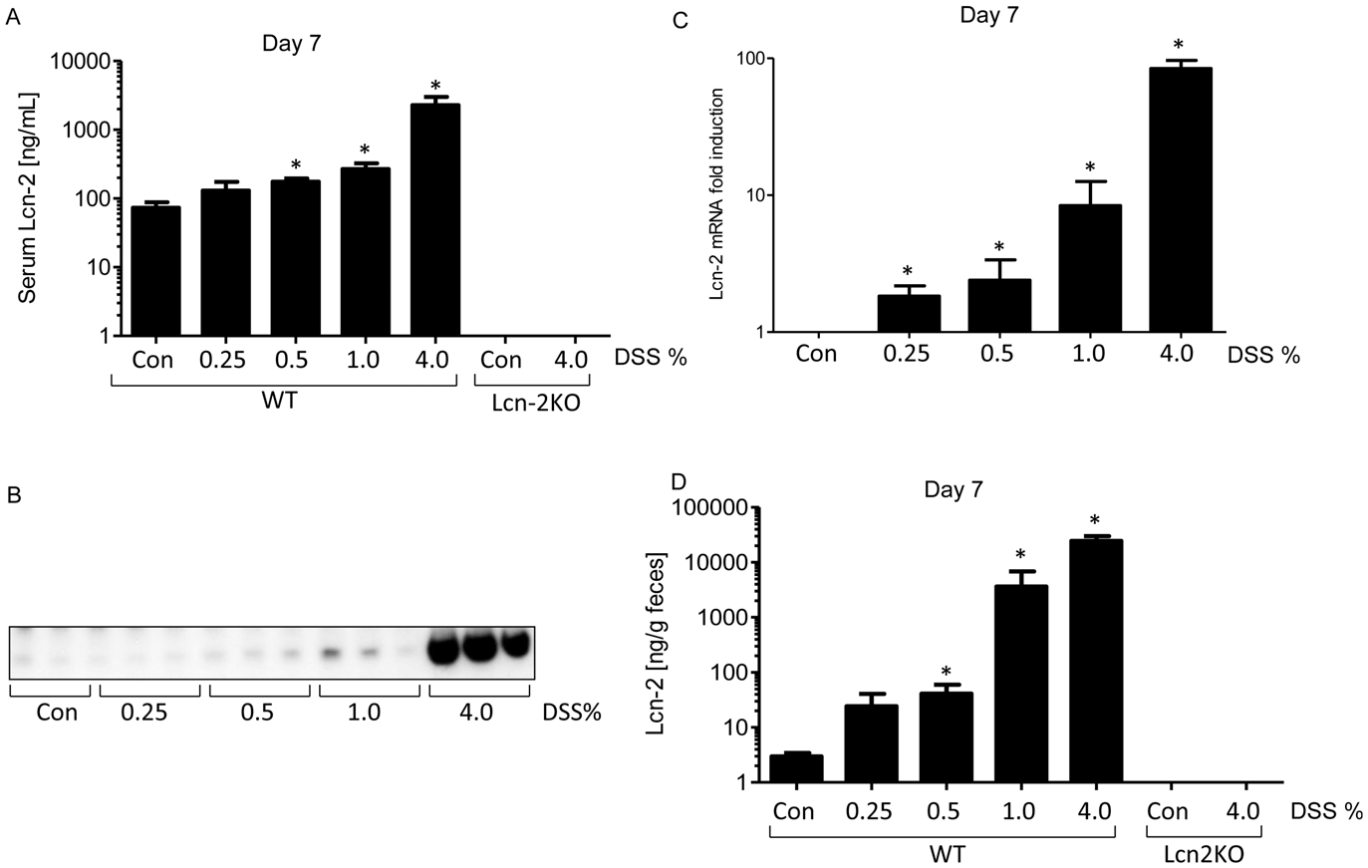
DSS induces Lcn-2 in a dose dependent manner. Serum Lcn-2 protein levels were analyzed by ELISA (**A**) and immunoblotting (**B**). Colonic Lcn-2 mRNA levels were analyzed by qRT-PCR (**C**) and fecal Lcn-2 levels were measured in feces at day 7 by ELISA (**D**). *p<0.05.

**Figure 4:**
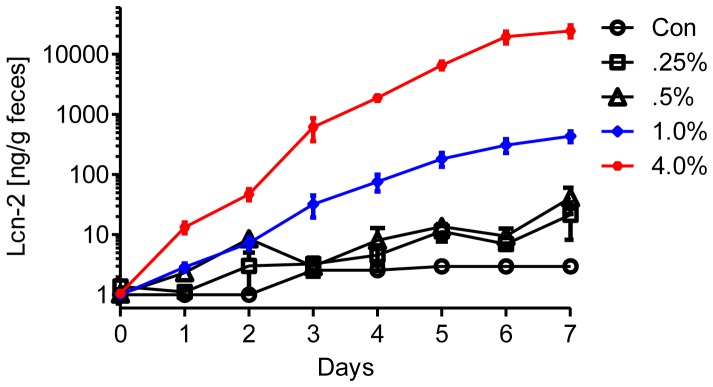
Substantial elevation of fecal Lcn-2 by DSS, in a time and dose dependent manner. Fecal Lcn-2 levels were measured in feces from day 0 through 7 by ELISA.

### Correlation of Gut Inflammation with Expression of Lcn-2

Levels of serum Lcn-2 have previously been used as a marker of inflammation [9], [11]. In accordance, we observed a roughly 20-fold increase in levels of serum Lcn-2 in response to 4% DSS and significant albeit lesser induction of this marker at lower DSS concentrations (**Figure 3A**). No Lcn-2 was detected in control sera or DSS-treated Lcn-2KO mice, verifying the specificity of the commercially purchased immunoreagents used for this assay (**Figure 3A**). We further verified our ELISA results by immunoblotting, which picked up substantial upregulation of serum Lcn-2 in mice given 4.0% and to a lesser extent in mice given 1.0% DSS (**Figure 3B**). These results verify that serum Lcn-2 can serve to detect robust intestinal inflammation but may have only a limited ability to detect low-grade intestinal inflammation. Consequently, we reasoned that measuring colonic expression of Lcn-2 might provide more sensitivity in response to DSS. In accordance with this notion, colonic expression of Lcn-2 mRNA as measured by qRT-PCR increased by 3, 5, 10 and 100 fold relative to the control group in the colons of mice given 0.25, 0.5, 1.0 and 4.0% DSS (**Figure 3C**). Given that much of the Lcn-2 produced by the intestine is made by intestinal epithelial cells which secrete the majority into the intestinal lumen [26], we hypothesized that DSS-induced intestinal Lcn-2 expression might be detectable in feces. Indeed, results presented in **figure 3D** demonstrated that fecal Lcn-2 was a sensitive and broadly dynamic marker of intestinal inflammation. Specifically, fecal Lcn-2 was upregulated by over 10-fold in response to 0.25% DSS and 10,000 fold in response to 4.0% DSS. No Lcn-2 was detected in feces of Lcn-2KO mice (control or DSS-treated), verifying the specificity of this assay. Next, to begin to investigate the utility of fecal Lcn-2 to monitor intestinal inflammation with minimal disturbance to mice, we measured the time course of induction of fecal Lcn-2 in response to DSS. As shown in **figure 4**, by day 1 fecal Lcn-2 levels significantly increased in all groups of mice given DSS, regardless of dose. By day 3, fecal Lcn-2 levels reached over 30- and 600-fold over controls in mice given 1.0 and 4.0% DSS, respectively, and continued to increase further in a dose- and time-dependent manner. Thus, fecal Lcn-2 is a sensitive marker capable of non-invasively detecting and monitoring low-grade inflammation. Moreover, fecal Lcn-2 has a broad dynamic range that allows discrimination between low-grade and robust intestinal inflammation.

**Figure 5:**
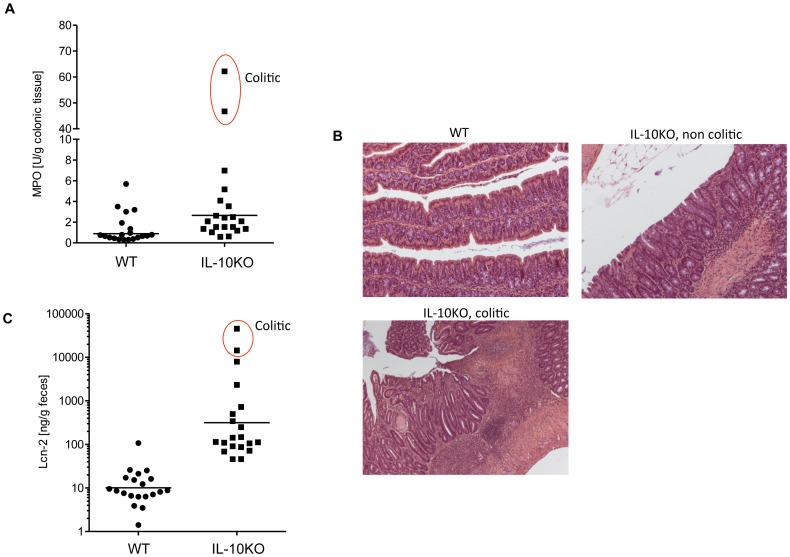
Fecal Lcn-2 marks the spontaneous colitis in IL-10 deficient mice. WT and IL-10 KO mice (n = 20) were analyzed for colonic MPO (**A**), H & E stained colon (**B**) and fecal Lcn-2 (**C**). Red circle indicate colitic mice.

### Fecal Lcn-2 Levels Marks the Severity of Spontaneous Colitis in IL-10 KO Mice

We next examined fecal Lcn-2 levels in IL-10 KO mice, which are prone to developing spontaneous colitis with the extent of colitis exhibiting great variability in different vivaria [27]. Eight-week-old female WT (BL6) and IL-10 KO mice were obtained from Jackson Labs, housed in specific pathogen free conditions, and monitored for colitis development for 12 weeks. After 12 weeks, fecal Lcn-2, colonic MPO and histology were evaluated. While there was some variability in MPO levels of WT mice, all 20 mice were within 5 standard deviations (SD) of the mean in accord with lack of evidence of colitis by histology. Analogously, fecal Lcn-2 levels varied in WT mice but all were within 5 SD of the mean. Thus, we viewed mice that had MPO of fecal Lcn-2 levels greater than 5 SD above the mean of WT mice as being elevated. Applying the cut-off derived from WT mice to IL-10-deficient mice revealed 2 mice elevated MPO (**figure 5A** **),** which were both exhibited histological signs of chronic inflammation with striking immune cell infiltration and epithelial erosion (**figure 5B**). These same 2 mice exhibited the highest levels of fecal Lcn-2, which were >3000 fold elevated relative to mean levels of WT mice thus indicating that measure of fecal Lcn-2 has ability to non-invasively identify severely colitic IL-10 deficient mice **(** **figure 5C** **)**. However, several other IL-10 deficient mice had fecal Lcn-2 greater than 5 SD above that of WT mice and all IL-10-deficient mice had levels of fecal Lcn-2 greater than the mean value of WT mice, indicating that fecal Lcn-2 levels also reflected the low-grade inflammation variably reflected mild immune cell infiltration. Thus fecal Lcn-2 is not only a sensitive marker for chemical induced colitis but also reflects severity of inflammation in a well-characterized immune-mediated colitis model.

**Figure 6:**
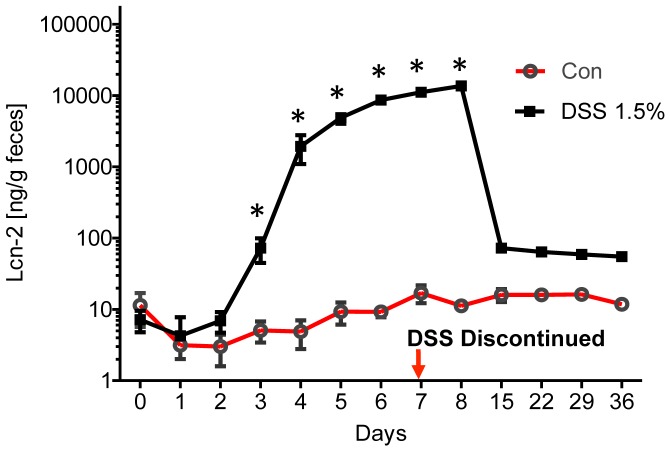
Substantial decrease in fecal Lcn-2 during mucosal healing. Mice were given 1.5% DSS in drinking water for 7 days (n = 5) and DSS was discontinued for 29 days. Fecal Lcn-2 levels were measured by ELISA (*p<0.05).

**Figure 7:**
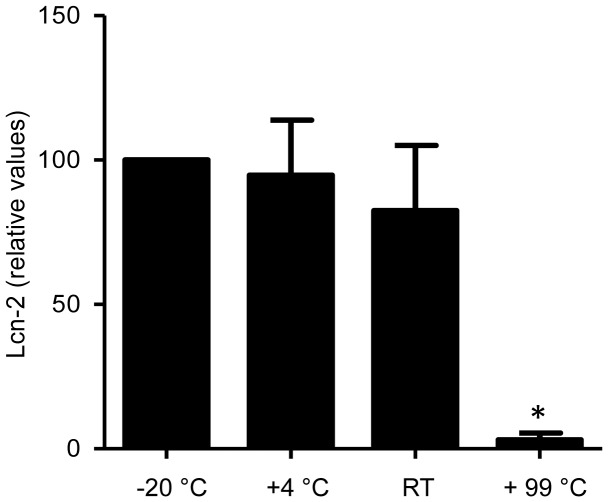
Fecal Lcn-2 is stable. Feces from 10 different mice were collected and split in four parts which were stored for 24 h at −20°C, 4°C, or room temperature (RT) or boiled for 1 h. Lcn-2 levels were measured by ELISA and expressed as values relative to those obtained from samples stored at −20°C. (*p<0.05).

### Substantial Decrease in Fecal Lcn-2 during Mucosal Healing

A necessary feature of a biomarker is rapid decrease in its levels on cessation of disease/inflammation. Therefore, we monitored fecal Lcn-2 levels during and after the termination of DSS in drinking water. As shown in **figure 6**, fecal Lcn-2 increased around 1000 fold following 7 days of DSS treatment. When DSS was discontinued, allowing mucosal healing, Lcn-2 levels dramatically decreased suggesting that fecal Lcn-2 exhibits this essential property.

### Fecal Lcn-2 is Stable

Another key feature of a valid biomarker is its stability during storage, freezing-thawing and transportation. To address the stability of fecal Lcn-2, we subjected fecal samples to a variety of conditions including freezing at −20°C, storing at 4°C or ambient room temperature (24°C) for 24 h or heating the samples to 99°C for 10 min and quantified fecal Lcn-2 in those samples by ELISA. The results indicated that no significant differences were observed after storage at 4°C, or at room temperature for 24 h when compared to −20°C, the temperature at which most clinical samples are stored prior to testing (**Figure 7**). However, heating the samples resulted in the loss of 95% ELISA reactivity. These results demonstrate that fecal Lcn-2 is more than stable enough to be used as an effective biomarker.

## Discussion

Lipocalin-2 (Lcn-2), originally appreciated as being highly abundant in neutrophils [8], has since been shown to be ubiquitously expressed and be highly induced in response to a wide variety of pro-inflammatory stimuli. Accordingly, levels of serum Lcn-2 have been shown to be elevated in both classic inflammatory conditions such as colitis and sepsis [11], [28] and, moreover, in states of low-grade inflammation such as metabolic syndrome, demonstrating that serum Lcn-2 could serve as a marker of inflammation [7]. Given that intestinal epithelial cells are one of the cell types in which Lcn-2 is highly induced, and that in these cells most Lcn-2 is secreted apically (i.e. luminally) [26], we hypothesized that fecal Lcn-2 might also mark intestinal inflammation thus serving as a non-invasive indicator of this state. Herein, we report that fecal Lcn-2, as assayed by ELISA, was a stable, highly sensitive and broadly dynamic marker of intestinal inflammation in mice. Specifically, fecal Lcn-2 was elevated by more than 10-fold in response to DSS concentrations that induced low-grade/sub-clinical inflammation and elevated by over 10,000-fold in response to DSS concentrations that induced histopathologically evident colitis. Such elevations in fecal Lcn-2 correlated well with intestinal mRNA levels of Lcn-2 and other pro-inflammatory genes, and thus fecal Lcn-2 can be viewed as reflecting the extent of activation of pro-inflammatory gene expression in the intestine. Thus, assay of fecal Lcn-2 can be used not only to identify mice with robust, i.e. histopathologically evident inflammation, but also to indicate low-grade or sub-clinical inflammation.

Importantly, in contrast to assessing inflammation via histopathology or by analysis of mRNA levels of pro-inflammatory genes or systemic cytokines, use of fecal Lcn-2 as a marker of inflammation is completely non-invasive and thus permits nearly constant monitoring (e.g. hourly, daily) of individual mice. Indeed, we used fecal Lcn-2 as a means to distinguish colitic and non-colitic toll-like receptor 5 (TLR5) deficient mice, thus enabling us to monitor the intestinal microbiota as colitis developed [29]. In addition, fecal Lcn-2 also serves as a marker of intestinal inflammation induced by the enteropathogen Adhesive-Invasive *E. coli* (AIEC), first isolated from a Crohn’s disease patient [29].

As sample acquisition and preparation is very simple and stable, we imagine a myriad of similar uses for this technique in basic intestinal research including the investigation of intestinal epithelial barrier dysfunction, increased gut bacterial burden/dysbiosis and enteropathogen colonization. All requisite reagents for fecal Lcn-2 assay via sandwich ELISA are commercially available at low cost (0.35 $ per sample) when compared to commercially existing stool biomarkers like calprotectin (6.70 $ per sample) [30], HMGB1 (12.5 $ per sample) [31], or lactoferrin (6.75 $ per sample) [32].

Thus, we expect that this technique will be widely adopted by other researchers resulting in improved experimentation, reduced numbers of mice per experiment, and concomitant reduced costs. While relative levels of serum Lcn-2 parallel those in feces, the extent of elevation of fecal Lcn-2 was much greater than that in serum likely reflecting that the fecal parameter is more indicative of conditions in the intestine. Consequently, it seems reasonable to speculate assay of fecal Lcn-2 in humans might prove to have clinical diagnostic value, perhaps as an early diagnostic marker that might be used to suggest an infection or an inflammatory bowel disease rather than the more common disease state of irritable bowel syndrome, which is not associated with robust inflammation. We anticipate that such questions will be a subject of future research.
